# Tricky Liver Case: Perihepatic Splenosis

**DOI:** 10.5334/jbsr.4153

**Published:** 2025-12-11

**Authors:** Ahmed Amine Dinia, Sandy Van Nieuwenhove, Qaid Ahmed Shagera

**Affiliations:** 1 Cliniques universitaires Saint Luc, Brussels, Belgium

**Keywords:** splenosis, 3D computed tomography, MRI, scintigraphy

## Abstract

*Teaching point:* Perihepatic splenosis should be considered in patients with a history of splenectomy presenting with a subdiaphragmatic mass that enhances similar to splenic tissue, to avoid unnecessary biopsy or surgery.

A 49‑year‑old man with a history of splenectomy 38 years earlier underwent contrast‑enhanced computed tomography (CT) as part of an evaluation for central hypogonadism. CT revealed a well‑defined, round, enhancing mass in the right subdiaphragmatic region (Figure 1A, arrow).

**Figure 1:**
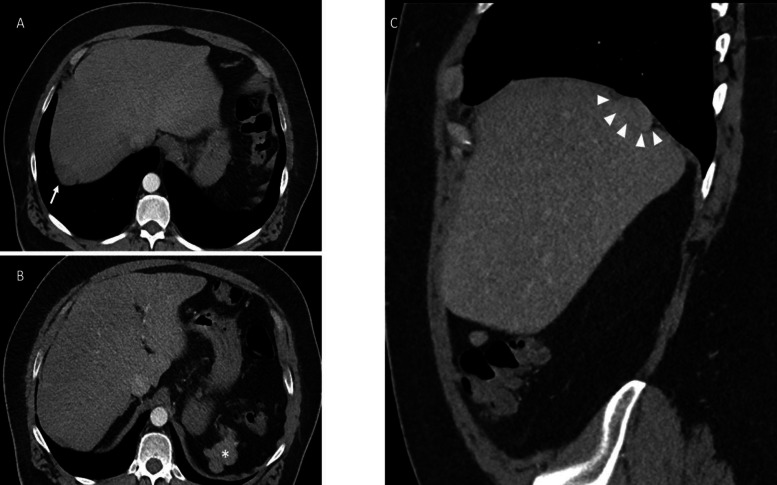
Incidentally perihepatic lesion in a 49‑year‑old man on contrast‑enhanced CT imaging. Axial **(A)** and sagittal images **(C)** showed a well‑defined enhancing mass that slightly deformed the diaphragmatic surface of the liver (white arrows) into a crescent shape (negative “embedded organ sign, white arrow heads) with dull edge (negative “beak sign”). Axial image **(B)** showed nodules into the left subdiaphragmatic space having the same density as the right peri hepatic lesion (*).

On sagittal reconstruction of the portal venous phase CT (Figure 1C), the lesion caused a slight impression on the diaphragmatic surface of the liver, creating a crescent‑shaped deformation (negative embedded organ sign) and a dull interface (negative beak sign) [1], suggesting an extrahepatic origin (Figure 1C, arrowheads). A left subdiaphragmatic polylobular nodule of similar density was also noted (Figure 1B, asterisk).

Magnetic resonance imaging (MRI) was performed for further characterization. The right subdiaphragmatic lesion appeared slightly hyperintense on fat‑suppressed T2‑weighted (Figure 2A, arrow) and T1‑weighted in‑phase images (Figure 2B) without signal drop on out‑of‑phase sequences (Figure 2C), excluding intralesional fat. A chemical shift artifact was visible between the lesion and the liver (Figure 2C, arrowheads). On axial T1 fat‑suppressed 3D GRE after intravenous gadolinium injection, the perihepatic lesion showed progressive, homogeneous enhancement across late arterial (Figure 2D) and delayed phase (Figure 2E), similar to the left subdiaphragmatic nodule (Figure 2F, asterisk).

**Figure 2:**
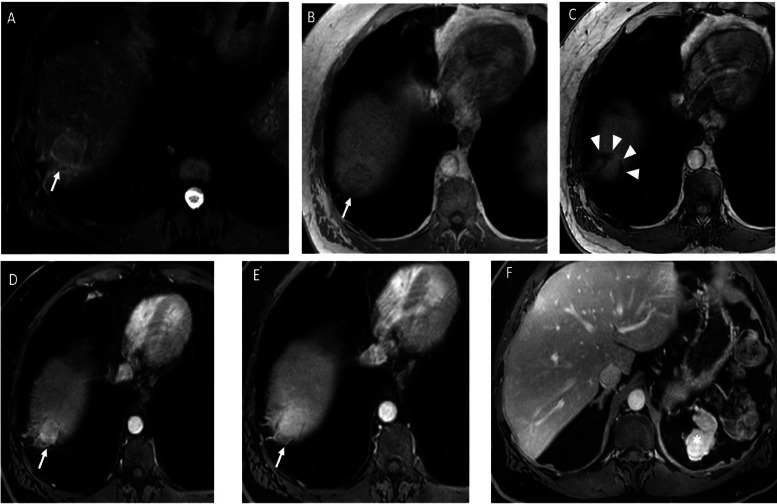
MRI images of the upper abdomen. Axial fat‑suppressed T2‑weighted image **(A)** showed that the right subdiaphragmatic lesion is slightly hyperintense (arrow). Axial T1‑in phase GRE image **(B)** and axial T1‑out‑of‑phase GRE image **(C)** showed that the lesion is slightly hypointense with no signal drop (arrow). Subdiaphragmatic fat is responsible of a fat chemical shift artifact between the lesion and the liver (arrowheads). Axial post‑contrast T1 mDixon MRI **(D, E, F)** showed progressive enhancement of the lesion (arrows), similar to the left subdiaphgramatic nodule (*).

To confirm the diagnosis, technetium‑99 m‑labeled heat‑damaged red blood cell (Tc‑99m RBC) scintigraphy was performed, confirming increased homogeneous radiotracer uptake within the right perihepatic (Figure 3A, arrow) and left subdiaphragmatic lesion (Figure 3B, curved arrow), consistent with splenic tissue.

**Figure 3:**
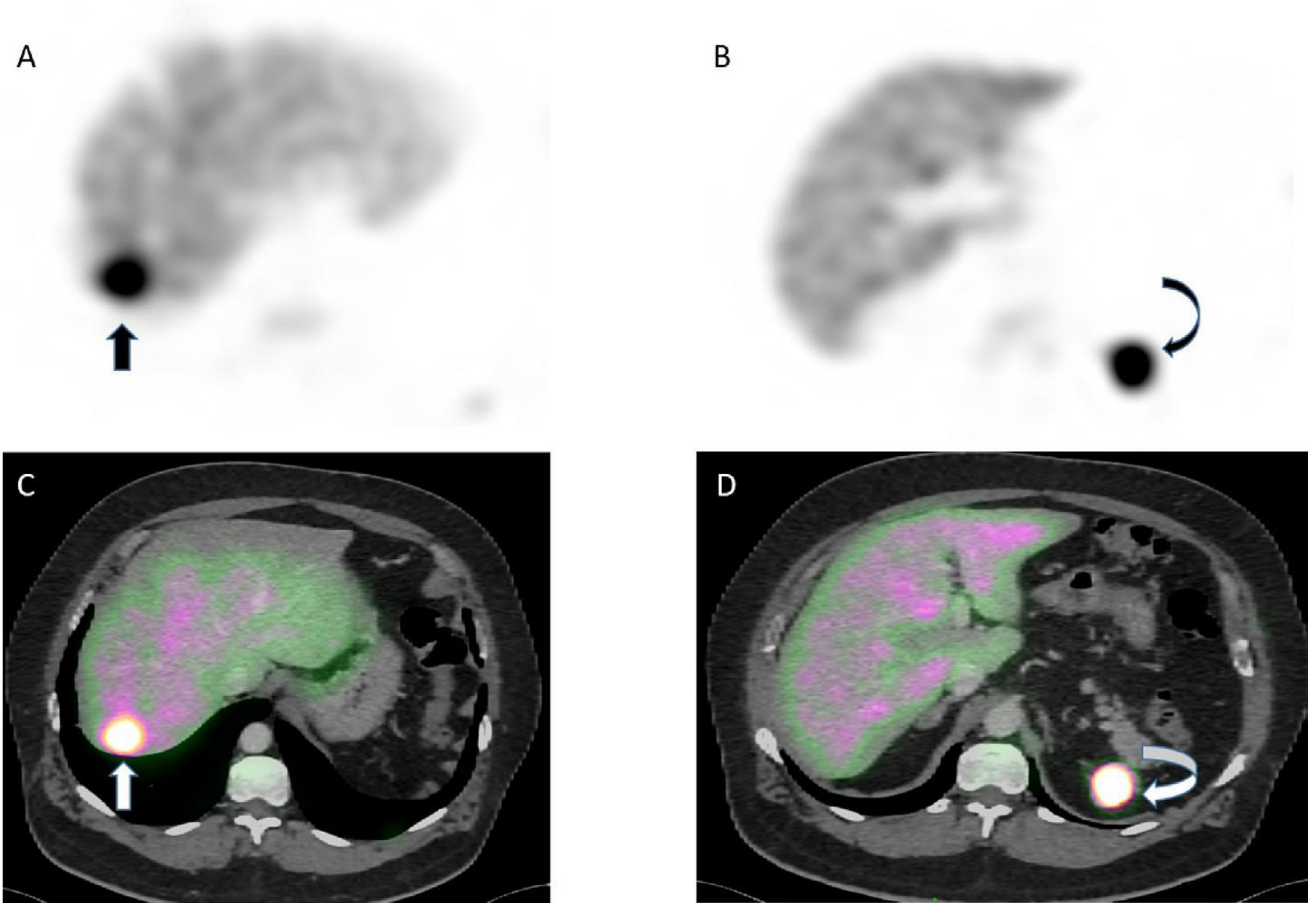
Axial Tc‑99m ‑ RBC scintigraphy **(A, B)** and fused with axial portal‑venous CT images **(C, D)** showed an increased homogenous radionuclide uptake in the perihepatic lesion (arrows) and in the left subdiaphragmatic mass corresponding to splenosis (curved arrows).

## Comment

Splenosis is a benign condition resulting from autotransplantation of splenic tissue after splenic rupture or surgery. The reported incidence among patients with traumatic splenic rupture ranges from 26% to 67%. It is usually discovered incidentally, even decades after splenectomy, and patients are typically asymptomatic. No treatment is required unless complications occur or diagnosis remains uncertain.

Lesions are well‑defined, round or lobulated, and often located in the left upper quadrant, but may occur anywhere in the peritoneal or extraperitoneal space. On CT and MRI, splenosis mimics normal splenic tissue in attenuation, signal intensity, and enhancement characteristics. The negative beak and negative embedded organ signs are valuable indicators of an extrahepatic origin.

Scintigraphy using Tc‑99m RBC is the most sensitive and specific diagnostic technique for detecting splenosis. Splenic tissue absorbs the Tc‑99m‑labeled damaged RBC demonstrating a homogeneous well‑defined uptake in ectopic splenic tissue.

Differential diagnosis includes peritoneal or perihepatic carcinomatosis and inflammatory hepatic adenoma. The history of splenectomy, negative beak and embedded organ signs, and enhancement identical to splenic tissue favored splenosis that has been confirmed by the Tc‑99m RBC scintigraphy. Carcinomatosis typically occurs in oncologic patients with ascites and multiple irregular nodules with delayed enhancement. Inflammatory adenoma is intrahepatic, occurs mostly in women with hepatic steatosis and shows hyperintensity on T2‑weighted images and persistent delayed enhancement.
